# Abingdon Academy of Medicine

**Published:** 1878-01-20

**Authors:** 


### ADVERTISEMENTS IN THE RECORD.

Several new and important advertisements may be seen for 1878, and certain of those formerly in are changed and enlarged.

Our subscribers are requested to examine them all. Much that is interesting and instructive may often be seen in advertisements. Men who advertise are generally active and enterprising in business, and will give prompt attention to orders. We hope our readers will patronize the houses which advertise in The Record, and ask their home druggists and merchants to do the same. Nearly all the leading cities are represented in our advertising department. We give below a list of the places and the business firms in each. For details see their respective advertisements:

Atlanta—The House of Pemberton, Samuels & Reynolds—wholesale and retail druggists—a new firm—pure drugs—active and reliable men.

Boston—Billings, Clapp & Co., manufacturing chemists of a high order—well established, safe and reliable.

New York—McKesson & Robbins, importers and jobbers of drugs, manufacturing chemists, etc. Large establishment—enterprising men—a house of the first order.

Reed & Carnrick, manufacturing pharmacists of a high order. A large and well managed business establishment. Stands among the best in the city.

Bellevue Hospital Medical College—Among the very best medical institutions in the United States.

Fremont, Ohio—Trommer Extract of Malt Co. —Liberal advertisers—active and enterprising. Their malt preparations are splendid.

Philadelphia—Wm. R. Warner & Co., manufacturing druggists—liberal advertisers. A large and enterprising establishment—live men—safe and reliable.

Bullock & Crenshaw, wholesale druggists, chemists and importers. An old and well established house—large establishment—active and safe men—extensive business.

John Wyeth & Bro., chemists and pharmaceutists—a very fine house—beautiful preparations—live men—large business.

Keasbey & Mattison, manufacturing chemists of high order—fine malt and numerous other preparations—large house—active men—safe, prompt and reliable.

Cincinnati, O.—Merrell Thorp & Lloyd, wholesale druggists and pharmacists—manufacturers of specific medicines—pure and reliable. A large and well established house—active and enterprising. At the very head of the list in the West.

St. Louis, Mo.—Aloe & Hernstein, surgical instruments and physician's supplies—one of the few St. Louis firms that advertise in the Eastern journals. A fine establishment—live and energetic business men.

Special Advertisements.—Among these we direct attention to the Liebig Beef Extract Co., Newburg, N. Y.; T. Coldon, proprietor, Baltimore.

Glycerite of Kephaline.—P. F. Biehl, Louisville, Ky.

Vitalized Phosphates—F. Crosby, chemist—Caswell, Hazzard & Co., agents, N. Y.

Cooper Truss Company, and others, which please see.

